# Compounding Impacts—Genomic Exploration of the Potential Effect of Domestic Introgression on Climate Adaptation in Atlantic Salmon (*Salmo salar*)

**DOI:** 10.1111/eva.70319

**Published:** 2026-09-29

**Authors:** Hallie Arno, Tony Kess, Steve Duffy, Benjamin Marquis, Samantha V. Beck, Sarah J. Lehnert, Ian A. Fleming, Ian R. Bradbury

**Affiliations:** ^1^ Department of Ocean Sciences Memorial University St. John's Newfoundland Canada; ^2^ Fisheries and Oceans Canada Northwest Atlantic Fisheries Centre St. John's Newfoundland Canada; ^3^ Great Lakes Forestry Center Natural Resources Canada Sault Ste Marie Ontario Canada; ^4^ Institute for Biodiversity and Freshwater Conservation University of the Highlands and Islands Inverness UK

**Keywords:** anthropogenic impacts, Atlantic salmon, climate change, domestication selection, population genomics

## Abstract

Predicting population, species, and community responses to climate change is critical for the successful management of wild ecosystems. However, climate change does not act alone. Interactions among climate change and other anthropogenic threats could significantly complicate predictions of global change yet have rarely been examined. *Salmo salar* (Atlantic salmon) are an increasingly at‐risk northern diadromous fish, exposed to multiple threats including a changing climate and introgression from net‐pen aquaculture escapees. Introgression from aquaculture escapees could alter wild salmon climate change responses through the introduction of maladaptive climate‐associated variation. Here we conducted a genome‐environment association analysis for wild Atlantic salmon throughout North America (*n* = 5208 samples) using a 220 K SNP array and explored differences between wild and aquaculture salmon at climate‐associated loci. Environmental associations suggest a polygenic basis to climate adaptation which includes a large chromosomal translocation, previously implicated in climate‐associated adaptation. Genomic differences between wild and domestic salmon generally increase with increasing geographic distance from the southern source location of North American salmon aquaculture. Wild salmon populations differed more genetically from aquaculture salmon (*F*_ST_ ~ two‐fold higher) at climate‐associated loci than genome‐wide, suggesting introgression may erode climate adaptation in the wild, and further amplify the negative impacts of climate change on these populations. The results suggest a more encompassing view of anthropogenic impacts which explicitly considers interactions among environmental and anthropogenic threats is needed to better understand and conserve wild populations in a changing world.

## 1 Introduction

Understanding and predicting the effects of interacting anthropogenic impacts is critical to the conservation of species, particularly in the face of climate change (Turner et al. 2020). The resilience of populations to environmental change is dependent in part on the extent of local adaptation, as well as the population's standing genetic variation (Barrett and Schluter 2008; Razgour et al. 2019). However, the impacts of climate change are unlikely to occur in isolation from other ecosystem changes, and the effects of multiple, interacting anthropogenic impacts can be complex (Côté et al. 2016; Crozier and Siegel 2025), reducing the long‐term adaptive capacity of wild populations through genetic changes that erode diversity (Derry et al. 2019; Munsch et al. 2022; Sgrò et al. 2011). The potentially complex interaction among anthropogenic threats raises the growing need for a broader approach to our understanding of climate change impacts but is often lacking in current climate change science.

The introduction of maladaptive genetic variation into wild populations through introgression of domestic escapees has been only minimally studied in relation to climate change (Crozier and Siegel 2025; Smith et al. 2022). Domestic species often differ morphologically, behaviorally, and genetically from their wild counterparts due to a mix of intentional and unintentional domestication selection likely leading to maladaptation in nature (Araki et al. 2007; Lavretsky et al. 2023; Leonard et al. 2013; Lynch and O'Hely 2001). In many ecosystems, domestic individuals significantly outnumber their wild counterparts (Smith et al. 2022; Sylvester et al. 2019), and genetic interactions between wild and intentionally or unintentionally released domestic individuals have been well documented in nature (Feulner et al. 2013; Glover et al. 2017; Kidd et al. 2009; Lavretsky et al. 2023; Mary et al. 2022). These genetic interactions between wild and domestic conspecifics have been repeatedly demonstrated to genetically and phenotypically alter wild populations (Randi 2008), often resulting in a reduction of fitness and population productivity in wild populations (Andrade et al. 2024; Wacker et al. 2021). This may be attributable to decreased tolerance to environmental stressors in domesticated species compared to their wild counterparts (e.g., Quezada‐Martinez et al. 2021; Smith et al. 2022), but it remains unknown to what degree introgression with domestic individuals can alter climate change responses in the wild.

*Salmo salar* (Atlantic salmon) is an anadromous salmonid widespread throughout the North Atlantic Ocean that is currently experiencing declines in abundance across much of its native range due to a suite of anthropogenic impacts (Forseth et al. 2017; Glover et al. 2017; Lehnert, Kess, et al. 2019). Atlantic salmon exhibit local adaptation throughout their range, where climate appears to be one of the primary drivers of population genetic and potentially adaptive differentiation (Bourret, Kent, et al. 2013; Horreo et al. 2011, 2019; Jeffery et al. 2017; Lehnert et al. 2023). Impacts of climate change on salmonids are widespread throughout their life cycle and include both direct temperature impacts (i.e., heat stress and changes to development rates) and indirect effects (i.e., habitat loss from altered stream flow, physiological stress from acidification, increased pathogen risk, and many others; Jonsson and Jonsson 2009; Thorstad et al. 2021). Concurrently, Atlantic salmon are also impacted throughout their range from hybridization with aquaculture escapees (Ford and Myers 2008; Naylor et al. 2005; Thorstad et al. 2008). Breeding programs in salmon aquaculture have resulted in changes to fitness‐linked traits (Fleming and Einum 1997; Perriman et al. 2022) and behaviors (Fleming and Einum 1997; Islam et al. 2020) that ultimately lead to reduced fitness and survival of domestic salmon in the wild (Fleming et al. 2000; McGinnity et al. 2003; Skaala et al. 2012, 2019; Sylvester et al. 2019). Models of aquaculture introgression predict that hybridization, even in small amounts, will lead to demographic decline in wild populations (Bradbury et al. 2019). To date, climate change and aquaculture have largely been studied independently as significant threats to the persistence of wild Atlantic salmon populations (but see McGinnity et al. 2009) leaving the potential for interaction among these threats unexamined.

Here, we explore the potential for domestic introgression to alter population responses to climate change in Atlantic salmon. Specifically, (1) we first examine the presence of genomic‐environmental associations (GEA) across the species range in the Northwest Atlantic using a 220 K SNP array spanning 131 wild populations; (2) we identify the genomic differences between wild and domestic salmon; and finally, (3) we evaluate overlap in genomic regions that are both climate associated and differ significantly between wild and domestic salmon to better understand the fitness impacts of introgression from escapees and climate change. By gaining a better understanding of the risks of the combined impacts of aquaculture introgression and climate change in this species, this work highlights the need for increased consideration of the interaction among anthropogenic threats to ultimately shape climate change impacts in wild populations, hence providing an integrated response to global change.

## 2 Methods

We investigated genomic differences between 131 wild populations (Figure 1A) and aquaculture (*n* = 440) Atlantic Salmon sampled throughout North America (*n* = 5648 fish sampled total). Aquaculture samples were obtained from the North American aquaculture strain spanning multiple producers in eastern Canada, presumably originating from the St. John River, New Brunswick before becoming part of a selective breeding program (Glebe 1988). All samples were previously genotyped (see Lehnert et al. 2020; Nugent et al. 2024; Wringe et al. 2018 for detailed sample genotyping methods) using a 220 K Axiom SNP array (Barson et al. 2015). We used SNPs that passed Axiom quality filters with minor allele frequencies of 0.05.

**FIGURE 1:**
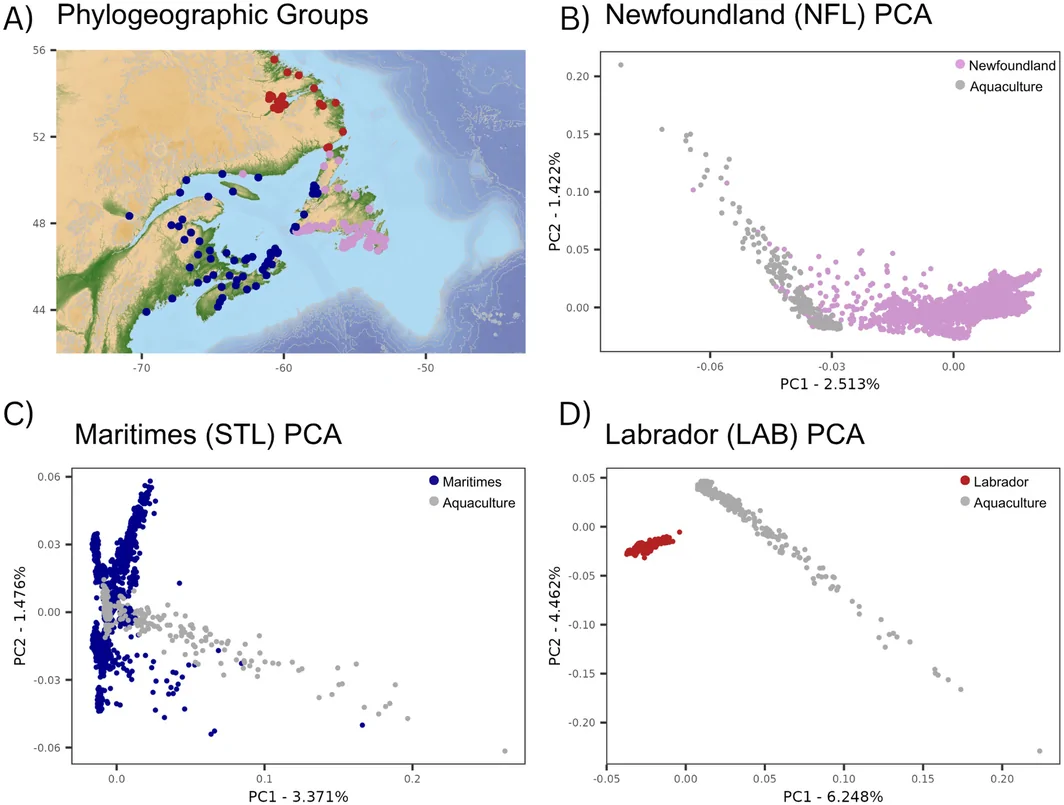
(A) Map of rivers sampled in North America. Color of markers represents phylogeographic group. Principle Components Analysis (PCA) showing differences in sampled SNPs between aquaculture fish (in gray) and fish from: (B) Newfoundland; (C) St. Lawrence and Maritimes; and (D) Labrador.

To identify SNPs strongly associated with climatic variation, we used a redundancy analysis (RDA) using the vegan package (Oksanen et al. 2024) for 26 climate variables compared to genomes of salmon range wide. We used 19 terrestrial bioclimatic variables from WorldClim (Fick and Hijmans 2017). This data is an average from 1970 to 2000 at a resolution of 30 s (~1 km^2^). In addition, we included sea surface temperature, salinity, and pH from MARSPEC (Sbrocco and Barber 2013) and Bio‐ORACLE (Assis et al. 2024) at the closest marine point to the river mouth during May through July to correspond with smolt outmigration (see Lehnert, Kess, et al. 2019). All environmental variables were scaled in R and a PCA was applied to scaled variables using the vegan package to extract correlations between variables (see Table S1 for PCA loadings). The top three PCs were used in the RDA, which was run for the entire geographic range of samples collected. RDA loadings were then mapped to SNP IDs and extracted to provide an absolute value of RDA loadings for each SNP. The SNPs in the highest 1% of absolute value of RDA loadings for each axis separately (RDA Axis 1, 2, and 3) were identified as being the most strongly associated with climate (“climate outliers”). Manhattan plots of *F*_ST_ values were created using the ggman package (Rajagopal 2016) for each phylogeographic group, and climate associated SNPs were highlighted. To visualize the proportion of SNPs that were in both the top 1% of *F*_ST_ and 1% of RDA loadings, *F*_ST_ was plotted against RDA loadings for each phylogeographic group and RDA axis.

Following Nugent et al. (2024), we investigated genomic differences at the scale of three distinct phylogeographic groups, as well as overall throughout North America. Three phylogeographic groups were identified due to distinct histories of post‐glacial recolonization and historical admixture from European populations, referred to as STL (Gulf of St. Lawrence and Maritime Canada), NFL (Newfoundland), and LAB (Labrador) (see Figure 1A). Genome‐wide differences (*F*_ST_) between wild and aquaculture fish North America‐wide and for each phylogeographic group were calculated using PLINK (Weir and Cockerham 1984). Within each group, SNPs were filtered using a minor allele frequency of 0.01 (Purcell et al. 2007) and locus‐specific *F*_ST_ was calculated. SNPs in the highest 1% of *F*_ST_ values were considered *F*_ST_ outliers and the most divergent between wild and aquaculture. Next, an unconstrained principal component analysis (PCA) using the R package pcadapt (Privé et al. 2020) was used to visualize the extent of differences between groups genome‐wide.

To further explore fine‐scale differences within individual populations, we then calculated *F*_ST_ between aquaculture and wild fish at the scale of each river. For each river, *F*_ST_ was calculated both genome‐wide and at climate outlier SNPs as identified by the RDA. These were plotted against each other for each river to visualize the extent to which climate‐associated *F*_ST_ scaled with genome‐wide *F*_ST_. Next, to explore putative functional differences, the overlap between SNPs in the highest 1% of *F*_ST_ (between wild and aquaculture) and the highest 1% of absolute RDA values were identified within the North‐America wide dataset. For these SNPs, we used bedtools intersect (Quinlan and Hall 2010) with a window size of 5000 bp to match to genes in the Ssal_v3.1 Atlantic Salmon annotated genome (GCA_905237065.2). We then conducted a gene ontology (GO) enrichment analysis using ShinyGO (v. 0.85 (Ge et al. 2020)) to link genes to functions and underlying molecular pathways.

## 3 Results

### 3.1 Genome‐Wide Differences

Clear genome‐wide differences between aquaculture fish and wild populations in each of the three phylogeographic groups were evident in the PCA and *F*_ST_ analyses (Figure 1B–D; Tables 1 and 2), and differences were strongest for Labrador (Figure 1D) and weakest for the St. Lawrence & Maritimes (Figure 1C) while Newfoundland was intermediate (Figure 1B) based on overlap in ordination space (PC1 and PC2) between wild and aquaculture populations. Mean *F*_ST_ values between aquaculture and each group reflect a similar pattern, with Labrador showing the highest divergence from the aquaculture (weighted *F*_ST_ = 0.084), with Newfoundland (weighted *F*_ST_ = 0.062) and St. Lawrence & Maritimes (weighted *F*_ST_ = 0.064) regions showing similar divergence (Table 1); however, Newfoundland had a slightly lower *F*_ST_ than the St. Lawrence & Maritimes genome wide. Throughout North America, the mean weighted *F*_ST_ between wild and aquaculture populations was 0.082.

**TABLE 1:** *F*_ST_ between aquaculture Atlantic salmon and wild populations overall and at each phylogeographic group (*LAB = Labrador, STL = St. Lawrence & Maritimes, NFL = Newfoundland*) for non‐climate associated SNPs.

Phylo group	Mean *F*_ST_	Weighted *F*_ST_	Fish, *N*	SNPs, *N*
North America	0.0710706	0.0820215	6344	142,887
STL	0.055361	0.0640029	2146	138,240
NFL	0.0546987	0.0622562	4143	143,139
LAB	0.0635102	0.0839742	836	149,139

**TABLE 2:** *F*_ST_ between aquaculture Atlantic salmon and wild populations overall and at each phylogeographic group (*LAB = Labrador, STL = St. Lawrence & Maritimes, NFL = Newfoundland*) for climate‐associated outliers.

Phylo group	Mean *F*_ST_	Weighted *F*_ST_	Fish, *N*	SNPs, *N*
North America	0.167718	0.186534	6344	4253
STL	0.0889554	0.099507	2146	3899
NFL	0.0975631	0.119181	4143	4043
LAB	0.145056	0.173488	836	4145

### 3.2 Environmentally‐Associated Differences

The RDA found that environmental factors represented a significant, though small, amount of genetic variation (adjusted *R* ^2^ = 0.038, *F*_3,5435_ = 68.203, *p* = 0.001). The first RDA axis represented 2.52% of variation, the second represented 1.06%, and the third represented 0.23%. The RDA separated phylogeographic groups and showed that Newfoundland and the Maritimes had more overlap than Labrador (Figure S1).

Climate‐associated SNPs often overlapped with very high *F*_ST_ SNPs between wild and aquaculture, or had a higher *F*_ST_ than average, especially when comparing *F*_ST_ between domestic‐wild and GEA outliers range‐wide (Figure 2). Across the North American range, *F*_ST_ between wild and aquaculture fish at climate‐associated SNPs was significantly higher (mean = 0.168, SD = 0.077) than *F*_*ST*_ values genome‐wide (mean = 0.071, SD = 0.039); (*t*_4254.4_ = −81.104, *p* < 2e‐16). This pattern remained consistent within each phylogeographic group (see Tables 1 and 2; Figure 4A). Although not all *F*_ST_ outliers overlapped with climate outliers, a higher proportion than expected did, where Newfoundland had 8.54% overlapping, and the Maritimes had 6.745% overlapping, and Labrador had 9.681% overlapping between the top 1% of climate‐associated and *F*_ST_ outliers across all three RDA axes (Figure 3 shows overlapping outliers for each phylogeographic group by RDA axis). When using all of the data for a North America wide analysis, 16.863% of SNPs overlapped. Within each phylogenetic group, the overlap between the top 1% climate outliers (across the first three RDA axes) and top 1% *F*_ST_ outliers was highest for Labrador (*n* = 546 SNPs), followed by the St. Lawrence & Maritimes (*n* = 355 SNPs) and Newfoundland (*n* = 473 SNPs).

**FIGURE 2:**
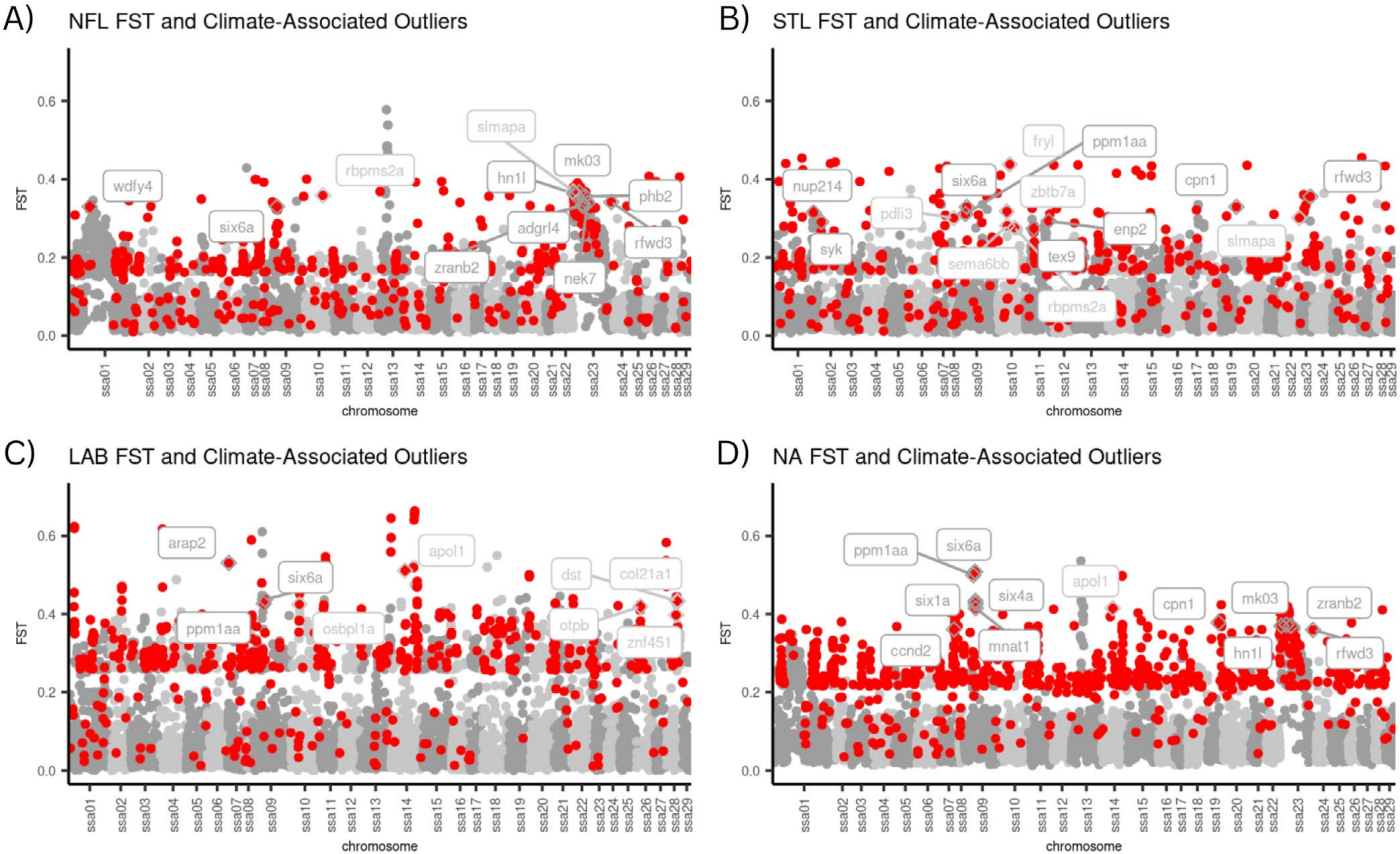
Manhattan plots showing *F*_ST_ by phylogeographic group (A–C; Newfoundland, St. Lawrence & Maritimes, and Labrador) and North America wide (D). Red highlighted points are climate‐associated (top 1% of RDA loadings). SNPs are annotated when they were within genes that are both climate‐associated and in the top 0.1% of FST values for each plot. A list of annotated genes can be found in Table S4.

**FIGURE 3:**
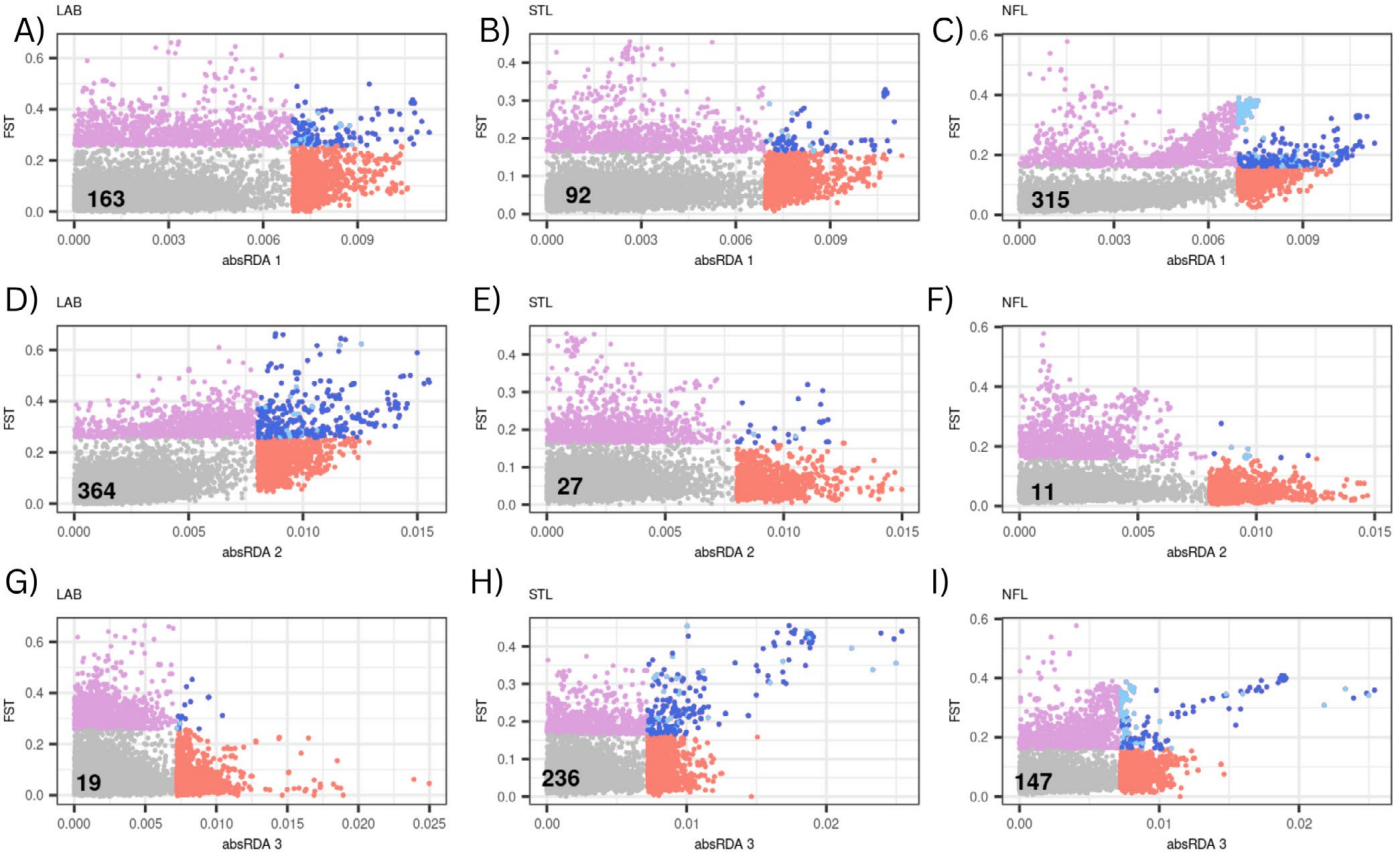
Plots of RDA versus *F*_ST_ between wild and aquaculture Salmon to highlight SNPs that are both RDA and *F*_ST_ outliers. Purple points are outliers from FST analysis, red points represent outliers on each RDA axis, and dark blue points are outliers on both (top 1%). Light blue points represent overlapping outliers that are on chromosomes 1 or 23. Gray points are a randomly chosen 5000 neutral SNPs. (A–C) show RDA1, (D–F) show RDA2, and (G–I) show RDA3. Panels are arranged in columns by a phylogeographic group (LAB = Labrador, STL = St. Lawrence & Maritimes, NFL = Newfoundland). The bold numbers represent the number of SNPs that overlap (total blue points).

We found enrichment of GEA associated divergence as well as high *F*_ST_ on chromosome 23 in the Newfoundland population (Figure 2A), a region associated with known intraspecific karyotype variation (Lehnert, Bentzen, et al. 2019). Newfoundland GEA outliers on RDA axes 1–3 had a higher proportion on chromosome Ssa01 than on other chromosomes (11.06%), suggesting a relation to this same previously described chromosomal translocation (Figure 3C,F,I; Figure S2).

At the scale of each river, mean *F*_ST_ at climate‐associated SNPs (mean = 0.094 ± 0.055) was higher than mean genome‐wide *F*_ST_ between aquaculture and wild comparisons (mean = 0.0461 ± 0.02) for nearly every river, with the exception of 16 rivers in the Maritimes. Of these 16 rivers, five are on Prince Edward Island, six are in Nova Scotia, three are in Quebec, and two are in New Brunswick (Kedgwick and Upper Northwest Miramichi). Overall, the difference between climate‐associated *F*_ST_ and genome‐wide *F*_ST_ between wild and aquaculture within each river sampled was greatest in Labrador and eastern Newfoundland (Figure 4B,C). The river with the greatest magnitude of difference was Crooked River in Labrador (climate‐associated *F*_ST_ = 0.167; genome‐wide *F*_ST_ = 0.059), and the river with the least climate‐associated difference in relation to genome‐wide difference was Morell River (Prince Edward Island) in the St. Lawrence & Maritimes group (climate‐associated *F*_ST_ = 0.024; genome‐wide *F*_ST_ = 0.027).

**FIGURE 4:**
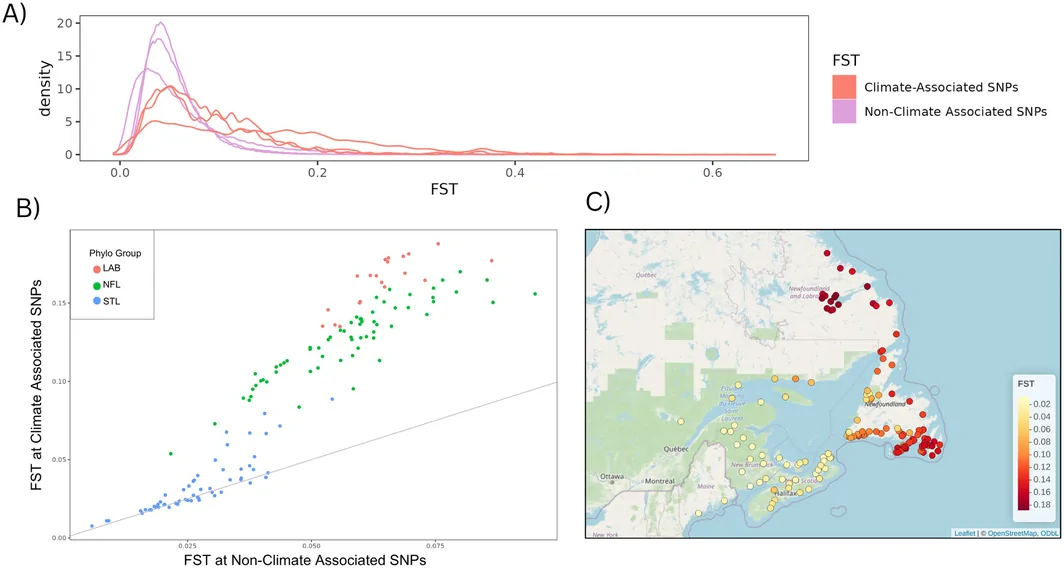
(A) Density plot of locus‐specific *F*_ST_ by phylogeographic group at climate associated SNPs compared to all SNPs. (B) Mean *F*_ST_ genome‐wide versus *F*_ST_ at climate associated SNPs between wild and aquaculture by river. Color represents phylogeographic group. (C) Map sample sites colored by *F*_ST_ at climate‐associated SNPs between wild and aquaculture.

A Gene Ontology (GO) Enrichment analysis revealed that overlapping SNPs that were both highly divergent from aquaculture and climate associated (*n* = 717) were enriched for GO annotations including ErbB signaling pathway, VEGF signaling pathway, C‐type lectin receptor signaling pathway, cellular senescence, focal adhesion, calcium signaling pathway, and multiple metabolic pathways (Figure S3, Tables S2 and S3).

## 4 Discussion

Understanding and predicting climate change impacts has become a significant focus of conservation and management efforts (Turner et al. 2020). Despite these efforts, consideration of how anthropogenic and genomic factors may interact to ultimately shape populations and species response to climate change is only beginning to be actively researched (see Layton et al. 2024). Theoretically, evaluating the complete threat landscape and accounting for interactions among threats should enhance understanding and predictions of climate change impacts on species and ecosystems. Here, our results suggest that wild populations of Atlantic salmon are adapted to local environments across the Northwest Atlantic and differ significantly from aquaculture salmon. Interestingly, wild salmon were more different from aquaculture salmon at climate‐associated genomic regions than genome‐wide. Our analyses indicate that these threats may compound one another, where domestic introgression into wild populations could constrain the ability of Atlantic salmon to respond to climate change due to the loss of local adaptation via introgression at climate‐associated loci. Our work builds directly on previous genomic studies of climate adaptation in Atlantic salmon (Bourret, Kent, et al. 2013; Jeffery et al. 2017; Sylvester et al. 2018; Verspoor and Jordan 1989; Watson et al. 2022), as well as studies documenting the genomic consequences of interbreeding with farm escapees (Bolstad et al. 2021; Glover et al. 2017; San Román et al. 2025). However, our study also highlights that explicit consideration of interactions among anthropogenic threats is needed if we are to understand and conserve wild populations under climate change.

### 4.1 Genome‐Environment Analysis

Our study provides the most comprehensive evaluation of genome‐environment associations for Atlantic salmon, incorporating high density genomic data with over 5000 individuals from 131 populations across the eastern North American range and within phylogeographic groups. We identified SNPs highly associated with 26 climate variables, in both the marine and freshwater environment, and these SNPs were distributed genome‐wide, suggesting a polygenic basis for climate adaptation. This polygenic signal associated with local adaptation to climate is consistent with results from previous studies on a diverse array of species, such as *Melanotaenia duboulayi* (Rainbowfish) (Smith et al. 2020), *Calinus hyperboreus* (copepods) (Davenport et al. 2026), *Fundulus heteroclitus* (Atlantic killifish) (Healy et al. 2018), *Haliotis rufescens* (Red abalone) (Griffiths et al. 2025), and for environmentally‐associated traits in many other species (reviewed in Bernatchez 2016). Previous studies using smaller genomic datasets have documented local adaptation to climatic conditions in Atlantic salmon at the landscape scale. Jeffery et al. (2017) found genomic clines associated with both geography and climate, particularly spring temperatures, as well as rainfall and snowfall, in Europe and North America, respectively. Bourret, Dionne, et al. (2013) detected signals of local adaptation to both climate factors and geological factors in North America and GO‐terms associated with outliers were related to growth. Earlier studies also revealed genetic structure influenced by the maximum summer temperatures, both in Europe and North America (Verspoor and Jordan 1989). Even at smaller spatial scales, temperature has been shown to be a driving factor for population structure in the species (Sylvester et al. 2018; Watson et al. 2022). In our analysis, we removed correlation between environmental variables by using principal components of environmental variables; therefore, we did not investigate which variables were most strongly associated. PCA loadings revealed that precipitation variables were highly represented on PC1, and air temperature on PC2 (Table S1). However, in the riverine environment, precipitation and water temperature are likely more closely correlated than in a terrestrial environment.

In our study, some of the climate‐associated SNPs were located within genes, including genes identified in previous studies of Atlantic Salmon and other species. One such example is the identification of *six6a* (Figure 2, Table S4) and genes in the six homeobox family exhibit association with age at maturity (Kurko et al. 2020), which has been shown to have a strong environmental component (Åsheim et al. 2023; Kess et al. 2024). In addition to individual SNPs associated with climate variables, we have found further support for a climate‐associated chromosomal translocation in southeastern Newfoundland. In parts of eastern Newfoundland such as Placentia Bay, post‐glacial secondary contact with European salmon resulted in an introgression of a chromosomal rearrangement between chromosomes Ssa01 and Ssa23 (Lehnert, Bentzen, et al. 2019; Nugent et al. 2024). Previous research has found this rearrangement is strongly correlated with temperature, providing further evidence for strong local adaptation to climatic conditions in these populations (Watson et al. 2022). Across the North American range, we found that chromosome Ssa01 had the highest number of climate outliers, further supporting this hypothesis (Figure S3).

### 4.2 Genomic Differences Between Wild and Aquaculture

At non‐climate associated SNPs, we observed large overlap in ordination space between wild and aquaculture fish in the St. Lawrence & Maritimes group, some overlap in Newfoundland, and no overlap in Labrador (Figure 1). Results from non‐climate associated *F*_ST_ values provided similar support (Table 1). SNPs with high *F*_ST_ values were found on every chromosome, even in the Maritimes where the stock likely originated, but chromosomes Ssa01, Ssa13, and Ssa23 each had a particularly high number of *F*_ST_ outliers. These large‐scale differences may have fitness consequences when aquaculture fish escape and hybridize with wild populations. In other species, alleles associated with domestication have shown to be selected against in the natural environment, suggesting that introgression from domestication results in a loss of fitness and local adaptation (Andrade et al. 2024). Within salmonids, domestication from aquaculture and hatcheries has been shown to result in changes to life‐history strategies (i.e., run timing), growth rate and size, and behavioral attributes, such as mating behaviors (Araki et al. 2008). Most studies find that domestication results in an overall loss of fitness in the wild due to reduced survival or reproductive output (Grant 2012; Tufto 2017). Indeed, numerous studies have shown fitness differences between wild and escaped aquaculture fish (e.g., Fleming et al. 2000; Kurko et al. 2020; McGinnity et al. 2003; Skaala et al. 2019; Sylvester et al. 2019). Between wild and aquaculture Atlantic Salmon, selection against domestication alleles in highly introgressed populations is predicted to lead to demographic decline (Bradbury et al. 2019; Castellani et al. 2018; Hindar et al. 2006). Our results suggest that part of this difference may be attributable to a loss of climate‐associated adaptation, which could lead to an increase in vulnerability as the climate changes.

### 4.3 Combined Impacts

At climate related outliers, divergence was highest in Labrador and lowest in the St. Lawrence & Maritimes group (Figure 4C). Though some of this pattern may be explained through isolation by distance from the original aquaculture source population (Saint John River), it is likely not the only factor since other rivers, notably on Prince Edward Island, are more similar to aquaculture fish at climate‐associated loci than the Saint John River and its tributaries. The Saint John River showed divergence between wild and aquaculture at climate‐associated SNPs, suggesting changes undergone by the domestication and selective breeding process. The risk of outbreeding depression increases with genetic differentiation (Edmands and Timmerman 2003), and our results suggest that the risk for outbreeding depression from aquaculture introgression is highest in Labrador, with the least risk on Prince Edward Island. Despite greater similarities between wild and aquaculture salmon in the St. Lawrence & Maritimes group, large climate associated divergence (weighted *F*_ST_ > 0.099 at climate‐associated loci) was still detected in our study, and introgression from escapees has been demonstrated to reduce local adaptation in this geographic region (Bourret et al. 2011). Additionally, at a finer spatial scale in this region, there are multiple genetic groups that vary in life history traits, environment, and genomic‐based adaptation, indicating the potential for heterogenous impacts within Maritimes rivers (Lehnert et al. 2023).

There have been demonstrated differences in climate adaptation associated with hatchery domestication in Atlantic salmon (McGinnity et al. 2009), and the impacts of domestic introgression have been shown to be dependent on environmental conditions in other salmonid species such as *Oncorhynchus mykiss* (rainbow trout) (Vandersteen et al. 2012). Previous studies have found that domestication may impact the ability to withstand variable conditions in fish. In *Oncorhynchus tshawytscha* (Chinook salmon), hatchery‐origin fish had a higher resting heart rate and smaller aerobic scope than their wild counterparts (Twardek et al. 2021), and highly domesticated *Hypomesus transpacificus* (Delta smelt) had lower thermal plasticity than less domesticated individuals (Griffiths et al. 2026). In terms of the ability to withstand higher temperatures, domesticated fish have been shown to have a lower critical thermal maximum than wild counterparts in salmonids (Carline and Machung 2001) and *Pimephales promelas* (Fathead minnows) (Hirakawa and Salinas 2020). The impacts of aquaculture introgression on fitness may be climate dependent and may increase the further populations are from the Maritimes, where the domesticated Atlantic salmon population originates, both in terms of physical distance and difference in climatic variables. Though the mechanisms for selection against climate‐associated alleles remain unclear, previous studies have demonstrated that faster growth, a trait that is selected for in the aquaculture environment, is negatively correlated with upper thermal tolerance (Debes et al. 2021). It may be that salmon in an aquaculture environment do not experience high thermal stress in the freshwater stage, while those in the wild do, leading to stronger selection in the wild with relaxed selection pressures in aquaculture.

The pathways that could be disrupted during introgression leading to a loss of local adaptation are highlighted in our gene‐ontology enrichment analysis (Figure S1) based on climate‐associated genes. These pathways were associated with cellular stress, focal adhesion, metabolism, and immunity. Cellular stress pathways identified here, such as epidermal growth factor receptor (ErbB) signaling, vascular endothelial growth factor (VEGF) signaling, and cellular senescence, have been shown to be associated with acute stress in Atlantic salmon (Madaro et al. 2023). VEGF has also been identified as an important pathway for oxygen supply in Atlantic salmon (Castro et al. 2013). Calcium signaling pathways were enriched in our analysis and have previously been identified in salmonids to be associated with osmoregulation and smoltification, the timing of which can be heavily environmentally driven (Beck et al. 2025; Domínguez et al. 2019). Pathways associated with immunity include C‐type lectin receptor and phosphatidylinositol signaling. Levels of phosphatidylinositol in Atlantic salmon cell membranes have been linked with diet and immune response to viruses in an aquaculture setting (Martinez‐Rubio et al. 2014). Immunity‐related pathways are particularly important to understand in relation to climate and aquaculture escapes, given that warmer temperatures can increase susceptibility to disease (Reverter et al. 2020) and pathogen transmission originating from aquaculture poses a threat to wild populations already (Krkosek et al. 2024; Thorstad et al. 2021). Pathways associated with metabolism include glycerolipid and glycerophospholipid metabolism and inositol phosphate metabolism. It is unsurprising that lipid metabolism differs between wild and aquaculture salmon, since it is likely under strong selection in aquaculture because of its link to fillet fat content (Harvey et al. 2024). Similarly, inositol phosphate metabolism may differ between wild and aquaculture because it is sometimes advised to supplement aquaculture salmonids with inositol (Shirmohammad et al. 2016). Many of our detected enriched pathways have also been found to be associated with age at maturity (Kess et al. 2024). Although gene ontology enrichments are a valuable hypothesis generating and exploratory tool, they should be interpreted with caution. Many of our enriched pathways had false discovery rate adjusted *p*‐values very close to 0.05, and relatively few genes found in each pathway (Table S3).

There are some potential limitations to the inferences made here, as we acknowledge that our analysis did not remove all wild samples potentially affected by aquaculture introgression. Given the proximity to of aquaculture to rivers sampled in New Brunswick and along the south coast of Newfoundland, it is possible that a small number of wild fish in our dataset from these regions have already been introgressed by aquaculture escapees, particularly on the south coast of Newfoundland (San Román et al. 2025; Wringe et al. 2018). Some rivers on the south coast of Newfoundland have a lower genome‐wide *F*_ST_ than would be expected compared to nearby rivers, likely due to introgression from nearby Aquaculture (Figure S4). Aquaculture pressure is strongest in southern Newfoundland, southern New Brunswick, and parts of Nova Scotia, while there is no salmon aquaculture in Labrador or Prince Edward Island (Atlantic Canada Fish Farmers Association, n.d.; Government of Newfoundland and Labrador 2026; Province of New Brunswick 2026; Province of Nova Scotia, n.d.). The populations geographically closest to aquaculture activity are likely at the highest risk of introgression (Glover et al. 2017; Keyser et al. 2018; San Román et al. 2025; Wringe et al. 2018). Although aquaculture pressure is low for northern Newfoundland and Labrador, although aquaculture escapees have been found more than 1000 km from the nearest aquaculture site in other countries (Bradbury et al. 2019; Glover et al. 2017; Hansen et al. 1997). In Canada, aquaculture escapees have generally been found within 300 km of aquaculture net pens (Bradbury et al. 2019; Morris et al. 2008). Another caveat is that, like many species, the fish in our dataset have already experienced some of the impacts of climate change. Our baseline climate dataset is an average from 1970 to 2000, before the most significant effects of climate change have been recorded. The rate of adaptation to climate is unknown in Atlantic salmon but has been shown to be detectable in only a few decades in other species (Adams et al. 2025). Without archival samples, it is impossible to know what the genomic architecture of populations would be without anthropogenic influence. However, our data still provides important insights into the existing standing genomic variation in populations that can be used to predict vulnerability to future climate change and introgression.

Although it is recognized that climate change and aquaculture introgression are both threats to wild Atlantic salmon populations (Dempson et al. 2024; Forseth et al. 2017; Thorstad et al. 2021), our results suggest that the interactions of such multiple threats should be considered when managing populations. Many areas with the greatest climate associated differences between local populations and domestic fish (i.e., southern Newfoundland) are near significant aquaculture activity, where large escapes have occurred in the past (Wringe et al. 2018). Our study highlights that predictions of the risk of introgression using genome‐wide comparisons between wild and aquaculture fish may underestimate impacts as the climate changes. We found that introgression from a domesticated population could exacerbate the impacts of climate change, and the loss to local climate adaptation from introgression may be greater than would be predicted based on the magnitude of genome‐wide differences. Understanding the combined impacts of these two anthropogenic threats is critical for conserving these populations in an increasingly human‐dominated world.

## Funding

This work was supported by Foundation for the Conservation of Atlantic Salmon, SAC‐2023‐02.

## Conflicts of Interest

The authors declare no conflicts of interest.

## Supporting information


**Figure S1:** RDA Biplot of PCs 1–3 of 26 environmental variables and 220K SNPs on all samples. Note that biplot arrows have been enlarged by a factor of 3 for image clarity.
**Figure S2:** GO Enrichment Plot of genes that are both climate associated and *F*_ST_ outliers between wild and aquaculture, using a false discovery rate (FDR) of 0.05.
**Figure S3:** Barplot of outliers by chromosome for all samples.
**Figure S4:** Mean FST between wild and aquaculture at the scale of each river.
**Table S1:** PCA loadings from climate variables used in RDA analysis.
**Table S2:** Genes in overlapping SNPs in the highest 1% of FST (between wild and aquaculture) and the highest 1% of absolute RDA values used in Gene Ontology Enrichment analysis.
**Table S3:** Enrichment of Genes in Pathways identified by GO analysis.
**Table S4:** Genes labelled on overall (NorthAm) and regional (*LAB = Labrador, STL = St. Lawrence & Maritimes, NFL = Newfoundland*) Manhattan plots in Figure 2.

## Data Availability

Combined genotypes and SNP positions are available on the Federal Research Data Repository. All code used for the analysis is free and publicly available (https://github.com/hallie‐arno/SalmonClimateAqaculture).
