# Supplementary material for: Compounding Impacts—Genomic Exploration of the Potential Effect of Domestic Introgression on Climate Adaptation in Atlantic Salmon (Salmo salar)
Source: Evol Appl. 2026 Sep 29;19(10):e70319. doi: 10.1111/eva.70319 (PMC13620849; doi:10.1111/eva.70319)
Supplement: Supplementary file 1 [file EVA-19-e70319-s001.docx]

## Supplements

*Figure S1: RDA Biplot of PCs 1-3 of 26 environmental variables and 220K SNPs on all samples. Note that biplot arrows have been enlarged by a factor of 3 for image clarity.*

*Figure S2: GO Enrichment Plot of genes that are both climate associated and F_ST_ outliers between wild and aquaculture, using a false discovery rate (FDR) of 0.05.*

*Figure S3: Barplot of outliers by chromosome for all samples.*

*Figure S4: Mean FST between wild and aquaculture at the scale of each river.*

Table S1: PCA loadings from climate variables used in RDA analysis.

| Variable | Description | PC1 | PC2 | PC3 | PC4 | PC5 |
| --- | --- | --- | --- | --- | --- | --- |
| Bio1 | Annual Mean Temperature | 0.17 | 0.28 | 0.137 | 0.126 | 0.072 |
| Bio2 | Mean Diurnal Range | -0.144 | 0.219 | 0.272 | -0.437 | -0.181 |
| Bio3 | Isothermality | 0.174 | 0.181 | 0.365 | -0.172 | -0.11 |
| Bio4 | Temperature Seasonality | -0.255 | 0.031 | -0.05 | -0.217 | -0.105 |
| Bio5 | Max Temperature of Warmest Month | -0.055 | 0.348 | 0.209 | -0.151 | 0.002 |
| Bio6 | Min Temperature of Coldest Month | 0.247 | 0.104 | 0.081 | 0.225 | 0.109 |
| Bio7 | Temperature Annual Range | -0.25 | 0.062 | 0.022 | -0.278 | -0.098 |
| Bio8 | Mean Temperature of Wettest Quarter | -0.167 | 0.037 | 0.309 | 0.246 | -0.53 |
| Bio9 | Mean Temperature of Driest Quarter | 0.203 | 0.219 | 0.072 | -0.094 | 0.255 |
| Bio10 | Mean Temperature of Warmest Quarter | 0.007 | 0.37 | 0.124 | 0.001 | -0.012 |
| Bio11 | Mean Temperature of Coldest Quarter | 0.235 | 0.152 | 0.111 | 0.187 | 0.101 |
| Bio12 | Annual Precipitation | 0.257 | 0.041 | -0.167 | -0.136 | -0.114 |
| Bio13 | Precipitation of Wettest Month | 0.233 | 0.096 | -0.237 | -0.268 | 0.008 |
| Bio14 | Precipitation of Driest Month | 0.257 | -0.005 | -0.137 | -0.083 | -0.212 |
| Bio15 | Precipitation Seasonality | -0.191 | 0.094 | -0.088 | -0.27 | 0.41 |
| Bio16 | Precipitation of Wettest Quarter | 0.245 | 0.069 | -0.218 | -0.193 | -0.058 |
| Bio17 | Precipitation of Driest Quarter | 0.258 | 0.032 | -0.133 | -0.099 | -0.171 |
| Bio18 | Precipitation of Warmest Quarter | 0.218 | -0.098 | -0.144 | -0.036 | -0.513 |
| Bio19 | Precipitation of Coldest Quarter | 0.256 | 0.054 | -0.099 | -0.192 | 0.032 |
| MS_sst05 | May SST at 5m | 0.026 | 0.303 | 0.053 | 0.082 | -0.037 |
| MS_sst06 | June SST at 5m | -0.023 | 0.326 | -0.113 | 0.011 | -0.059 |
| MS_sst07 | July SST at 5m | 0.038 | 0.285 | -0.063 | 0.415 | 0.034 |
| MS_sss05 | May salinity at 5m | 0.167 | -0.248 | 0.281 | -0.049 | 0.129 |
| MS_sss06 | June salinity at 5m | 0.199 | -0.205 | 0.238 | -0.05 | 0.11 |
| MS_sss07 | July salinity at 5m | 0.227 | -0.152 | 0.215 | -0.039 | 0.056 |
| BO_ph | pH | 0.145 | -0.226 | 0.43 | -0.141 | 0.021 |

Table S2: Genes in overlapping SNPs in the highest 1% of FST (between wild and aquaculture) and the highest 1% of absolute RDA values used in Gene Ontology Enrichment analysis.

| Gene | Symbol | Chr | Position (Mbp) | Description and Source |
| --- | --- | --- | --- | --- |
| LOC106599824 | agap3 | 3 | 32.834413 | ArfGAP with GTPase domain, ankyrin repeat and PH domain 3 [ZFIN;Acc:ZDB-GENE-110927-1] |
| PLCC | agpat3 | 16 | 63.638304 | 1-acylglycerol-3-phosphate O-acyltransferase 3 [ZFIN;Acc:ZDB-GENE-040426-2765] |
| LOC106590041 | ARHGAP12 | 29 | 3.380326 | Rho GTPase activating protein 12 [HGNC Symbol;Acc:HGNC:16348] |
| LOC106603582 | arrb2a | 4 | 52.712072 | arrestin, beta 2a [ZFIN;Acc:ZDB-GENE-030131-29] |
| LOC106571696 | asb2a.1 | 15 | 45.88764 | ankyrin repeat and SOCS box containing 2a, tandem duplicate 1 [ZFIN;Acc:ZDB-GENE-030131-5355] |
| LOC106613679 | atcaya | 10 | 18.374156 | ATCAY kinesin light chain interacting caytaxin a [ZFIN;Acc:ZDB-GENE-070705-185] |
| LOC106572032 | atp2b3a | 15 | 79.911077 | ATPase plasma membrane Ca2+ transporting 3a [ZFIN;Acc:ZDB-GENE-040718-174] |
| LOC106577124 | atrnl1a | 18 | 25.260521 | attractin-like 1a [ZFIN;Acc:ZDB-GENE-081028-60] |
| AUH | auh | 1 | 165.255039 | AU RNA binding protein/enoyl-CoA hydratase [ZFIN;Acc:ZDB-GENE-040801-95] |
| BTBD7 | btbd7 | 15 | 46.185144 | BTB (POZ) domain containing 7 [ZFIN;Acc:ZDB-GENE-030131-4477] |
| LOC106567954 | C2CD2 | 13 | 79.729132 | C2 calcium dependent domain containing 2 [HGNC Symbol;Acc:HGNC:1266] |
| LOC106607754 | C6orf58 | 15 | 36.125959 | chromosome 6 open reading frame 58 [HGNC Symbol;Acc:HGNC:20960] |
| LOC106584031 | cachd1 | 23 | 9.540836 | cache domain containing 1 [ZFIN;Acc:ZDB-GENE-100819-3] |
| CCND2 | ccnd2 | 7 | 45.872944 | G1/S-specific cyclin-D2 [NCBI gene (formerly Entrezgene);Acc:100195486] |
| LOC100286410 | col2a1a | 15 | 66.729847 | collagen, type II, alpha 1a [ZFIN;Acc:ZDB-GENE-980526-192] |
| LOC106583928 | cramp1 | 23 | 1.665042 | cramped chromatin regulator homolog 1 [ZFIN;Acc:ZDB-GENE-030131-8255] |
| LOC106610228 | CSNK1D | 1 | 91.856622 | casein kinase 1 delta [HGNC Symbol;Acc:HGNC:2452] |
| DHRS7 | dhrs7 | 9 | 23.743674 | dehydrogenase/reductase (SDR family) member 7 [ZFIN;Acc:ZDB-GENE-060825-21] |
| LOC106570084 | dnajc8 | 14 | 58.267396 | DnaJ (Hsp40) homolog, subfamily C, member 8 [ZFIN;Acc:ZDB-GENE-030131-66] |
| LOC106611717 | drp2 | 9 | 71.384057 | dystrophin related protein 2 [ZFIN;Acc:ZDB-GENE-070228-4] |
| LOC106609440 | DYRK4 | 7 | 45.943788 | dual specificity tyrosine phosphorylation regulated kinase 4 [HGNC Symbol;Acc:HGNC:3095] |
| LOC106570299 | EEPD1 | 14 | 74.791116 | endonuclease/exonuclease/phosphatase family domain containing 1 [HGNC Symbol;Acc:HGNC:22223] |
| LOC106569200 | elovl1a | 14 | 26.412728 | ELOVL fatty acid elongase 1a [ZFIN;Acc:ZDB-GENE-041010-66] |
| LOC106576814 | emx2 | 18 | 8.025453 | empty spiracles homeobox 2 [ZFIN;Acc:ZDB-GENE-990415-54] |
| LOC106613682 | eps15l1a | 10 | 18.505869 | epidermal growth factor receptor pathway substrate 15 like 1 [HGNC Symbol;Acc:HGNC:24634] |
| LOC106570353 | eya3 | 14 | 78.239875 | EYA transcriptional coactivator and phosphatase 3 [ZFIN;Acc:ZDB-GENE-060503-864] |
| LOC106582452 | FARP1 | 21 | 49.56445 | FERM, ARH/RhoGEF and pleckstrin domain protein 1 [HGNC Symbol;Acc:HGNC:3591] |
| LOC106609463 | FGF23 | 7 | 45.890822 | fibroblast growth factor 23 [HGNC Symbol;Acc:HGNC:3680] |
| LOC106609464 | FGF23 | 7 | 45.900945 | fibroblast growth factor 23 [HGNC Symbol;Acc:HGNC:3680] |
| LOC106609473 | FGF6 | 7 | 45.907224 | fibroblast growth factor 6 [HGNC Symbol;Acc:HGNC:3684] |
| LOC106567522 | FSTL4 | 13 | 59.739558 | follistatin like 4 [HGNC Symbol;Acc:HGNC:21389] |
| LOC106583904 | gdpd3a | 23 | 2.598692 | glycerophosphodiester phosphodiesterase domain containing 3a [ZFIN;Acc:ZDB-GENE-040426-2279] |
| LOC106586638 | gja5a | 25 | 33.705297 | gap junction protein, alpha 5a [ZFIN;Acc:ZDB-GENE-040407-3] |
| LOC106602381 | glra3 | 4 | 16.569883 | glycine receptor, alpha 3 [ZFIN;Acc:ZDB-GENE-020402-1] |
| LOC106570361 | hivep1 | 14 | 78.65089 | HIVEP zinc finger 1 [ZFIN;Acc:ZDB-GENE-070912-512] |
| HSPBAP1 | hspbap1 | 21 | 40.632847 | hspb associated protein 1 [NCBI gene;Acc:445320] |
| LOC106562145 | igdcc4 | 11 | 16.341286 | immunoglobulin superfamily, DCC subclass, member 4 [ZFIN;Acc:ZDB-GENE-080327-32] |
| LOC106582292 | ikzf2 | 21 | 37.657545 | IKAROS family zinc finger 2 [ZFIN;Acc:ZDB-GENE-100922-169] |
| LOC106571433 | itpkb | 15 | 40.831929 | inositol-trisphosphate 3-kinase B [ZFIN;Acc:ZDB-GENE-041014-166] |
| LOC106612967 | KIRREL3 | 9 | 135.097234 | kirre like nephrin family adhesion molecule 3 [HGNC Symbol;Acc:HGNC:23204] |
| MK03 | mk03 | 23 | 2.629164 | Mitogen-activated protein kinase 3 [NCBI gene (formerly Entrezgene);Acc:100380511] |
| MNAT1 | mnat1 | 9 | 23.906182 | MNAT1 component of CDK activating kinase [ZFIN;Acc:ZDB-GENE-041010-203] |
| LOC106603292 | msi2a | 4 | 47.403193 | musashi RNA-binding protein 2a [ZFIN;Acc:ZDB-GENE-030826-25] |
| MTAP | mtap | 8 | 17.726078 | methylthioadenosine phosphorylase [NCBI gene (formerly Entrezgene);Acc:100195118] |
| LOC106613675 | mydgf | 10 | 18.32215 | myeloid-derived growth factor [ZFIN;Acc:ZDB-GENE-040718-183] |
| LOC106570010 | necap1 | 14 | 62.876687 | NECAP endocytosis associated 1 [ZFIN;Acc:ZDB-GENE-040426-1682] |
| LOC106562216 | NEDD4 | 11 | 20.757097 | NEDD4 E3 ubiquitin protein ligase [HGNC Symbol;Acc:HGNC:7727] |
| NFIL3 | nfil3 | 1 | 165.315827 | nuclear factor, interleukin 3 regulated [NCBI gene;Acc:445509] |
| NFIL3 | nfil3-6 | 6 | 34.036864 | nuclear factor, interleukin 3 regulated, member 6 [ZFIN;Acc:ZDB-GENE-040704-63] |
| NR1H4 | nr1h4 | 15 | 79.947671 | nuclear receptor subfamily 1, group H, member 5 [ZFIN;Acc:ZDB-GENE-080403-3] |
| LOC106609526 | nudt4b | 7 | 49.651425 | nudix (nucleoside diphosphate linked moiety X)-type motif 4b [ZFIN;Acc:ZDB-GENE-040912-79] |
| P2RY8 | p2ry8 | 16 | 75.966304 | P2Y purinoceptor 8 [NCBI gene (formerly Entrezgene);Acc:100195699] |
| LOC106583913 | pax10 | 23 | 2.308416 | paired box 10 [ZFIN;Acc:ZDB-GENE-081022-10] |
| LOC106577482 | pcdh7b | 18 | 52.810432 | protocadherin 7b [ZFIN;Acc:ZDB-GENE-081104-299] |
| PDIA5 | pdia5 | 21 | 41.097798 | protein disulfide isomerase family A member 5 [NCBI gene (formerly Entrezgene);Acc:100194934] |
| PDL1 | pdl1 | 13 | 102.33665 | Programmed cell death 1 ligand 1 [NCBI gene (formerly Entrezgene);Acc:100196322] |
| PGCP | pgcp | 2 | 12.182813 | Plasma glutamate carboxypeptidase [NCBI gene (formerly Entrezgene);Acc:100195413] |
| PLCG2 | plcg2 | 16 | 34.598575 | phospholipase C, gamma 2 [ZFIN;Acc:ZDB-GENE-030131-9532] |
| LOC106580958 | rabgef1l | 20 | 59.351693 | RAB guanine nucleotide exchange factor (GEF) 1, like [ZFIN;Acc:ZDB-GENE-040426-813] |
| LOC106582448 | RAP2A | 21 | 49.040532 | RAP2A, member of RAS oncogene family [HGNC Symbol;Acc:HGNC:9861] |
| LOC106605639 | rbm42 | 5 | 67.694845 | RNA binding motif protein 42 [NCBI gene;Acc:445360] |
| LOC106568222 | rhof | 13 | 91.119134 | ras homolog family member F [ZFIN;Acc:ZDB-GENE-050522-280] |
| ROR2 | ror2 | 1 | 165.332309 | receptor tyrosine kinase-like orphan receptor 2 [ZFIN;Acc:ZDB-GENE-060427-5] |
| LOC106573506 | RORA | 16 | 28.087568 | RAR related orphan receptor A [HGNC Symbol;Acc:HGNC:10258] |
| LOC106583918 | rras | 23 | 2.127494 | RAS related [ZFIN;Acc:ZDB-GENE-041010-217] |
| LOC106604578 | sgpl1 | 1 | 51.559465 | sphingosine-1-phosphate lyase 1 [ZFIN;Acc:ZDB-GENE-070410-24] |
| LOC106568190 | sgsm1b | 13 | 92.132981 | small G protein signaling modulator 1b [ZFIN;Acc:ZDB-GENE-091117-39] |
| LOC106570009 | si:ch211-232m10.6 | 14 | 62.89237 | si:ch211-232m10.6 [ZFIN;Acc:ZDB-GENE-060503-879] |
| LOC106605589 | si:ch211-79k12.1 | 2 | 24.674807 | si:ch211-79k12.1 [ZFIN;Acc:ZDB-GENE-080303-9] |
| LOC106582352 | slc15a2 | 21 | 40.614089 | solute carrier family 15 member 2 [NCBI gene;Acc:556684] |
| LOC106610249 | SLC16A3 | 1 | 91.820817 | solute carrier family 16 member 3 [HGNC Symbol;Acc:HGNC:10924] |
| LOC106609368 | slc26a4 | 7 | 45.185922 | solute carrier family 26 member 4 [ZFIN;Acc:ZDB-GENE-060503-544] |
| SLC38A6 | slc38a6 | 9 | 23.980342 | solute carrier family 38 member 6 [NCBI gene;Acc:336741] |
| LOC106567511 | stk10 | 13 | 58.704311 | serine/threonine kinase 10 [NCBI gene;Acc:394108] |
| LOC106611118 | stxbp6 | 9 | 27.006186 | syntaxin binding protein 6 (amisyn) [ZFIN;Acc:ZDB-GENE-040625-31] |
| SYK | syk | 1 | 165.168727 | spleen tyrosine kinase [NCBI gene (formerly Entrezgene);Acc:100380388] |
| LOC106583946 | taok2a | 23 | 3.240314 | TAO kinase 2a [ZFIN;Acc:ZDB-GENE-030131-9416] |
| LOC106572516 | tfcp2 | 15 | 88.363341 | transcription factor CP2 [ZFIN;Acc:ZDB-GENE-030131-2523] |
| LOC106568223 | tmem120b | 13 | 91.105243 | transmembrane protein 120B [NCBI gene;Acc:798580] |
| LOC106593970 | tmem260 | 1 | 28.354502 | transmembrane protein 260 [ZFIN;Acc:ZDB-GENE-030131-4198] |
| LOC106571367 | TPD52L1 | 15 | 37.114568 | tpd52 like 1 [ZFIN;Acc:ZDB-GENE-050522-121] |
| UBR7 | ubr7 | 15 | 46.311554 | ubiquitin protein ligase E3 component n-recognin 7 [NCBI gene (formerly Entrezgene);Acc:100194874] |
| VASH2 | vash2 | 15 | 40.506096 | vasohibin 2 [ZFIN;Acc:ZDB-GENE-041014-355] |
| WDR11 | wdr11 | 18 | 2.896953 | WD repeat domain 11 [HGNC Symbol;Acc:HGNC:13831] |
| ZN576 | zn576 | 5 | 69.264333 | Zinc finger protein 576 [NCBI gene (formerly Entrezgene);Acc:100194986] |
| ENP2 |  | 11 | 84.198641 | ectonucleoside triphosphate diphosphohydrolase 2b [ZFIN;Acc:ZDB-GENE-061013-2] |
| LOC106570083 |  | 14 | 58.28783 | heterogeneous nuclear ribonucleoprotein R [ZFIN;Acc:ZDB-GENE-040426-2766] |
| LOC106580758 |  | 20 | 51.929218 | si:ch211-137a8.4 [ZFIN;Acc:ZDB-GENE-030131-3742] |

**Table S3: Enrichment of Genes in Pathways identyified by GO analysis.**

| Enrichment FDR | nGenes | Pathway Genes | Fold Enrichment | Pathway | Genes |
| --- | --- | --- | --- | --- | --- |
| 0.00353388 | 4 | 117 | 17.6737109 | Path:sasa04625 C-type lectin receptor signaling pathway | syk mk03 plcg2 rras |
| 0.04119157 | 2 | 58 | 17.8260705 | Path:sasa00561 Glycerolipid metabolism | agpat3 |
| 0.04119157 | 2 | 62 | 16.6760014 | Path:sasa00562 Inositol phosphate metabolism | itpkb plcg2 |
| 0.04119157 | 7 | 1089 | 3.32294978 | Path:sasa01100 Metabolic pathways | mtap itpkb plcg2 agpat3 gdpd3a |
| 0.04119157 | 3 | 216 | 7.17994505 | Path:sasa04020 Calcium signaling pathway | itpkb atp2b3a plcg2 |
| 0.04119157 | 2 | 71 | 14.5621421 | Path:sasa04070 Phosphatidylinositol signaling system | itpkb plcg2 |
| 0.04119157 | 3 | 168 | 9.23135793 | Path:sasa04218 Cellular senescence | ccnd2 mk03 rras |
| 0.04119157 | 2 | 59 | 17.5239337 | Path:sasa04370 VEGF signaling pathway | mk03 plcg2 |
| 0.04119157 | 3 | 198 | 7.83266733 | Path:sasa04510 Focal adhesion | ccnd2 col2a1a mk03 |
| 0.04344033 | 2 | 78 | 13.2552832 | Path:sasa04012 ErbB signaling pathway | mk03 plcg2 |
| 0.045501 | 2 | 84 | 12.3084772 | Path:sasa00564 Glycerophospholipid metabolism | agpat3 |
| 0.07023814 | 3 | 294 | 5.27506167 | Path:sasa04010 MAPK signaling pathway | mk03 rras taok2a |
| 0.07657588 | 2 | 121 | 8.544728 | Path:sasa04140 Autophagy-animal | mk03 rras |
| 0.0789197 | 2 | 128 | 8.07743819 | Path:sasa04371 Apelin signaling pathway | mk03 rras |
| 0.08916409 | 2 | 142 | 7.28107104 | Path:sasa04261 Adrenergic signaling in cardiomyocytes | mk03 atp2b3a |
| 0.09922021 | 2 | 156 | 6.62764159 | Path:sasa04068 FoxO signaling pathway | ccnd2 mk03 |
| 0.15077009 | 2 | 204 | 5.06819651 | Path:sasa04810 Regulation of actin cytoskeleton | mk03 rras |
| 0.1781092 | 1 | 40 | 12.9239011 | Path:sasa00565 Ether lipid metabolism | gdpd3a |
| 0.17948544 | 1 | 43 | 12.0222336 | Path:sasa00270 Cysteine and methionine metabolism | mtap |
| 0.17948544 | 1 | 45 | 11.4879121 | Path:sasa04340 Hedgehog signaling pathway | ccnd2 |
| 0.045501 | 2 | 84 | 12.3084772 | Path:sasa00564 Glycerophospholipid metabolism | agpat3 |
| 0.07023814 | 3 | 294 | 5.27506167 | Path:sasa04010 MAPK signaling pathway | mk03 rras taok2a |
| 0.07657588 | 2 | 121 | 8.544728 | Path:sasa04140 Autophagy-animal | mk03 rras |
| 0.0789197 | 2 | 128 | 8.07743819 | Path:sasa04371 Apelin signaling pathway | mk03 rras |
| 0.08916409 | 2 | 142 | 7.28107104 | Path:sasa04261 Adrenergic signaling in cardiomyocytes | mk03 atp2b3a |
| 0.09922021 | 2 | 156 | 6.62764159 | Path:sasa04068 FoxO signaling pathway | ccnd2 mk03 |
| 0.15077009 | 2 | 204 | 5.06819651 | Path:sasa04810 Regulation of actin cytoskeleton | mk03 rras |
| 0.1781092 | 1 | 40 | 12.9239011 | Path:sasa00565 Ether lipid metabolism | gdpd3a |
| 0.17948544 | 1 | 43 | 12.0222336 | Path:sasa00270 Cysteine and methionine metabolism | mtap |
| 0.17948544 | 1 | 45 | 11.4879121 | Path:sasa04340 Hedgehog signaling pathway | ccnd2 |

Table S4: Genes labelled on overall (NorthAm) and regional (*LAB=Labrador, STL=St. Lawrence & Maritimes, NFL=Newfoundland*) Manhattan plots in Figure 2.

| Chromosome | SNP | Position | Gene | Group |
| --- | --- | --- | --- | --- |
| ssa01 | AX-86958517 | 49562757 | wdfy4 | NFL |
| ssa09 | AX-87142373 | 23856316 | six6a | NFL |
| ssa10 | AX-87594018 | 70780362 | rbpms2a | NFL |
| ssa22 | AX-87125895 | 60989550 | slmapa | NFL |
| ssa23 | AX-86919763 | 36365735 | rfwd3 | NFL |
| ssa23 | AX-86944514 | 3926458 | zranb2 | NFL |
| ssa23 | AX-86983773 | 2630008 | mk03 | NFL |
| ssa23 | AX-87030070 | 5375800 | nek7 | NFL |
| ssa23 | AX-87165759 | 1647682 | hn1l | NFL |
| ssa23 | AX-87486464 | 4763361 | phb2 | NFL |
| ssa23 | AX-87709750 | 3894868 | adgrl4 | NFL |
| ssa07 | AX-87464177 | 8499707 | arap2 | LAB |
| ssa09 | AX-87142373 | 23856316 | six6a | LAB |
| ssa09 | AX-87583737 | 23787529 | ppm1aa | LAB |
| ssa10 | AX-86980632 | 25030934 | osbpl1a | LAB |
| ssa14 | AX-86939607 | 34472894 | apol1 | LAB |
| ssa26 | AX-87300404 | 4886722 | otpb | LAB |
| ssa28 | AX-87103565 | 27358857 | dst | LAB |
| ssa28 | AX-87460449 | 27510338 | col21a1 | LAB |
| ssa28 | AX-87746256 | 27243783 | znf451 | LAB |
| ssa01 | AX-87185999 | 138945663 | nup214 | STL |
| ssa01 | AX-87187831 | 165197623 | syk | STL |
| ssa08 | AX-87336891 | 6459076 | pdli3 | STL |
| ssa09 | AX-87142373 | 23856316 | six6a | STL |
| ssa09 | AX-87583737 | 23787529 | ppm1aa | STL |
| ssa10 | AX-87203378 | 23747901 | sema6bb | STL |
| ssa10 | AX-87266824 | 21960363 | zbtb7a | STL |
| ssa10 | AX-87594018 | 70780362 | rbpms2a | STL |
| ssa10 | AX-87690048 | 32531997 | fryl | STL |
| ssa11 | AX-87254893 | 20692780 | tex9 | STL |
| ssa11 | AX-87443239 | 84202128 | enp2 | STL |
| ssa19 | AX-87067819 | 66942139 | cpn1 | STL |
| ssa22 | AX-87125895 | 60989550 | slmapa | STL |
| ssa23 | AX-86919763 | 36365735 | rfwd3 | STL |
| ssa07 | AX-87447468 | 45880317 | ccnd2 | NorthAm |
| ssa09 | AX-86991079 | 23921737 | mnat1 | NorthAm |
| ssa09 | AX-87005183 | 23905281 | six1a | NorthAm |
| ssa09 | AX-87142373 | 23856316 | six6a | NorthAm |
| ssa09 | AX-87522853 | 23913892 | six4a | NorthAm |
| ssa09 | AX-87583737 | 23787529 | ppm1aa | NorthAm |
| ssa14 | AX-86939607 | 34472894 | apol1 | NorthAm |
| ssa19 | AX-87067819 | 66942139 | cpn1 | NorthAm |
| ssa23 | AX-86919763 | 36365735 | rfwd3 | NorthAm |
| ssa23 | AX-86944514 | 3926458 | zranb2 | NorthAm |
| ssa23 | AX-86983773 | 2630008 | mk03 | NorthAm |
| ssa23 | AX-87165759 | 1647682 | hn1l | NorthAm |
